# Bilateral carotid artery stenosis causes unexpected early changes in brain extracellular matrix and blood-brain barrier integrity in mice

**DOI:** 10.1371/journal.pone.0195765

**Published:** 2018-04-12

**Authors:** Jill M. Roberts, Michael E. Maniskas, Gregory J. Bix

**Affiliations:** 1 Sanders-Brown Center on Aging, University of Kentucky, Lexington, Kentucky, United States of America; 2 Department of Neuroscience, University of Kentucky, Lexington, Kentucky, United States of America; 3 Department of Neurosurgery, University of Kentucky, Lexington, Kentucky, United States of America; 4 Department of Neurology, University of Kentucky, Lexington, Kentucky, United States of America; Hungarian Academy of Sciences, HUNGARY

## Abstract

Bilateral carotid artery stenosis (BCAS) is one experimental model of vascular dementia thought to preferentially impact brain white matter. Indeed, few studies report hippocampal and cortical pathology prior to 30 days post-stenosis; though it is unclear whether those studies examined regions outside the white matter. Since changes in the blood-brain barrier (BBB) permeability precede more overt brain pathology in various diseases, we hypothesized that changes within the BBB and/or BBB-associated extracellular matrix (ECM) could occur earlier after BCAS in the hippocampus, cortex and striatum and be a precursor of longer term pathology. Here, C57Bl/6 mice underwent BCAS or sham surgeries and changes in the BBB and ECM were analyzed by collagen IV (vascular basement membrane component), α5 integrin (marker of endothelial activation), claudin-5 and occludin (tight junction proteins), Evans blue (permeability marker), Ki-67 (cell proliferation marker), and GFAP and CD11b (glial cell markers) immunohistochemistry after 14 days. Significant changes in markers of cerebrovascular integrity and glial activation were detected, not only in the striatum, but also in the hippocampus and cortex. In conclusion, this study demonstrates for the first time that changes in the BBB/ECM occur shortly after BCAS and within multiple brain regions and suggests such changes might underlie the gradual development of BCAS non-white matter pathology.

## Introduction

Vascular pathology is the second leading cause of dementia behind Alzheimer’s disease and will likely become more prominent in patients in the coming years [1]. Vascular dementia (VaD) is thought to develop from chronic cerebral hypoperfusion, which with time leads to white matter lesions and cognitive impairment. Bilateral carotid artery stenosis (BCAS) is a commonly used model of cerebral hypoperfusion-induced cognitive impairment, using micro-coils wrapped around the arteries to decrease blood flow to the brain [2]. Many rodent studies have shown this reduced carotid artery blood flow causes alterations within the corpus callosum and caudoputamen within 30 days. However, the extent of hippocampal and cortical pathology remains unclear, as investigators have either not observed change in these regions and therefore do not report it or few studies have actually examined regions outside of the white matter.

The blood-brain barrier (BBB) consists of several layers of protection to restrict the infiltration of various substances between the blood and brain parenchyma and an important component of the BBB is the extracellular matrix (ECM), which provides structural and biochemical support to the endothelial cells. Failure of the BBB/ECM is a significant occurrence in the development and progression of various diseases, such as stroke and multiple sclerosis [3–7]. In the mouse BCAS model of chronic cerebral hypoperfusion, studies have shown significant BBB leakage within the corpus callosum [8, 9] and correlate this breakdown with the resulting white matter lesions. However, little is known about the extent of BBB breakdown in non-white matter regions at early time points. Therefore, we hypothesized that changes within the BBB or the ECM could occur earlier than 30 days after BCAS in the cortex, hippocampus, and striatum (caudoputamen) and be a precursor of longer term pathology in these regions.

To examine this, we employed the mouse BCAS model for 7 or 14 days and looked at immunohistochemical changes in components of the BBB, ECM, glial cells and cell proliferation within all three brain regions.

## Materials and methods

### Animals

The experimental protocol was approved by the Institutional Animal Care and Use Committee of the University of Kentucky (protocol #2014–1287) and experiments were performed in accordance with the Guide for the Care and Use of Laboratory Animals of the National Institutes of Health and reported according to the ARRIVE guidelines (S1 File). All analyses were performed in a blinded fashion and animals were randomly assigned (via Research Randomizer online) to treatment groups. All surgeries were performed under ketamine/xylazine anesthesia and all efforts were made to minimize suffering and were performed within the animal facility during the animals light cycle. All mice were housed in a climate-controlled room on a 14/10 hour light/dark cycle (respectively) and food and water were provided *ad libitum*.

### Bilateral carotid artery stenosis (BCAS) model

To induce prolonged cerebral hypoperfusion, 3 month old male C57Bl/6 mice (total N = 13 sham and 29 BCAS; Jackson Labs) were anesthetized with a ketamine/xylazine cocktail and a midline incision was made to expose both common carotid arteries (CCA). Two 4–0 silk sutures were placed under the proximal and distal portions of the left CCA and were used to gently lift the artery for placement of the micro-coil (0.18 mm diameter; Waken B Technology Company Limited, Japan) by rotating it around the artery just below the carotid bifurcation. Immediately following, a second micro-coil was placed on the right CCA. The incision was then sutured and the animals were allowed to recover for 7 or 14 days. Sham animals underwent the same procedure without the placement of the micro-coils.

### Post-surgical monitoring and euthanasia methods

Immediately following surgery, the mice received an injection (s.c.) of Buprenorphine-SR-Lab (1 mg/ml; ZooPharm) analgesic and were placed in a warm recovery cage. Mice were monitored until fully awake, mobile and interested in food/water and were then placed in their home cage in the animal facility. Mice underwent daily monitoring for signs of sickness or discomfort, including infection at incision site, lethargy, disinterest in food/water, weight loss and seizure. Mice exhibiting these symptoms were immediately euthanized via cervical dislocation/decapitation under ketamine/xylazine anesthetic. For this study, an N = 6 (BCAS group only) either experienced a seizure following surgery (N = 4) or died before termination of the study due to unknown causes (N = 2). At termination of the study (day 7 or 14), mice were euthanized by cervical dislocation/decapitation under ketamine/xylazine anesthetic.

### Immunohistochemistry

On post-stenosis days 7 and 14, the mice were euthanized and the brains were immediately removed, flash frozen and stored at -20°C. Brains were cut into 20 μm sections using a cryostat and directly mounted onto slides. Sections were fixed with ice cold acetone/methanol (50:50 mixture) prior to incubating in blocking buffer (5% BSA in 1xPBS with 0.1% Triton X-100) for one hour at room temperature. The sections were then incubated overnight at 4°C with the following primary antibodies: Collagen IV (Abcam; 1:250), CD49e (α5; BD Pharmingen; 1:250), FITC-conjugated tomato-lectin (Vector Labs; 1:200), Claudin-5 (Abcam; 1:250), GFAP (Life Technologies; 1:250), CD11b (AbD Serotec; 1:250), Ki-67 (Abcam; 1:250), PECAM-1 (Millipore; 1:250), and Oligodendrocyte Specific Protein (Abcam; 1:250). Sections were washed and incubated with a secondary antibody (AlexaFluor 568 or 488; Life Technologies; 1:500) for one hour at room temperature. Slides were then washed and coverslipped with fluorescent mounting media (Vector Labs) and images were captured using an Eclipse Ti microscope/DS-Ri1 CCD color camera and NES analysis software (Nikon).

**Fig 1:**
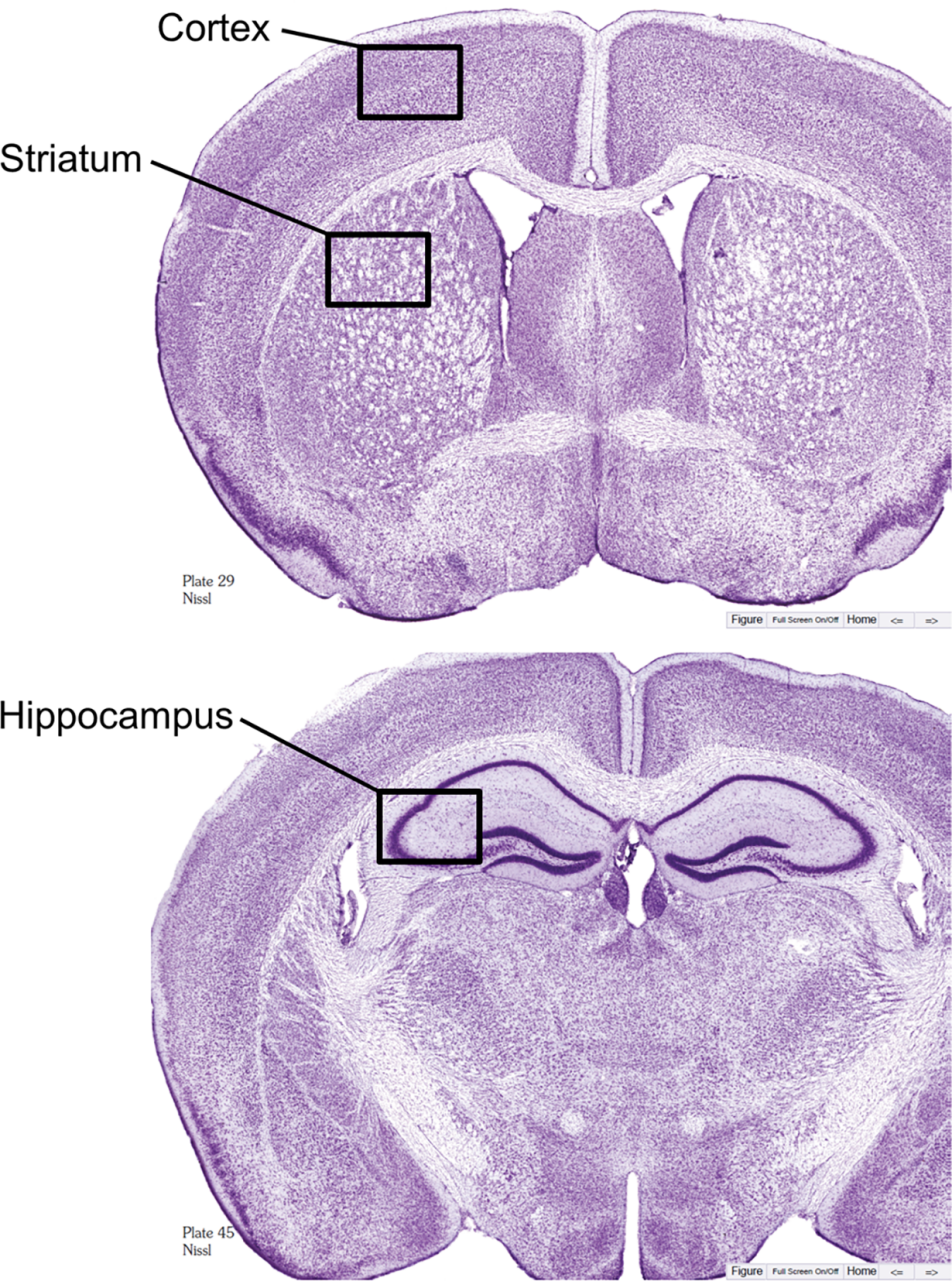
Histological diagrams. Location of images (black box) for immunohistochemical analysis of the cortex, striatum and hippocampus. Modified Nissl sections from The Mouse Brain by Franklin & Paxinos (3^rd^ edition).

Images were taken within the cortex (watershed region), striatum (caudoputamen) and hippocampus (CA2-3 region) (Fig 1) from sham and BCAS-treated mice. Three images per region per mouse were then analyzed for the number of stain-specific positive pixels using Photoshop (Adobe). Images were converted to grayscale, adjusted to a set threshold equal to the antibody staining and the number of pixels calculated. The data were averaged per group and are presented as number of positive pixels.

Co-localization analysis was performed using Image J. Briefly, merged images were color separated (red and green) and a threshold equal to the antibody staining was set. Each color was selected and added to a selection manager which created a new image of overlapping areas between the two channels. The area of the individual channel selection and the overlapping image selection was measured. The data are presented at the percent of Ki-67-positive cells that are also stain-specific positive (PECAM-1, GFAP, CD11b, OSP).

### Evans blue extravasation

On post-stenosis day 14, sham and BCAS-treated mice received intravenous injections of Evans blue dye (0.2 ml of a 2% solution in saline; Sigma), which circulated for 30 minutes. The mice then underwent transcardial perfusion of 1xPBS and the brains were removed and immediately flash frozen and stored at -20°C. Brains were cut into 20 μm sections using a cryostat, mounted onto slides and coverslipped with fluorescent mounting media (Evans blue dye fluoresces red). Alternatively, sections were counterstained with FITC-conjugated tomato-lectin to visualize blood vessels. Images and analysis were obtained as mentioned above.

### Gene expression

On post-stenosis day 14, brains were removed and cut into 2 mm thick sections. The sections were cut to isolate the cortex and the striatum of each hemisphere, and tissue was homogenized in Trizol (Life Technologies). RNA was extracted according to the instructions of the RNA extraction kit manufacturer (PureLink RNA Mini kit; ThermoFisher Scientific). RNA was converted to cDNA using a high capacity cDNA reverse transcription kit (Applied Bioscience). Occludin and 18S (control) genes (Life Technologies) were analyzed using Real-time PCR (ViiA7; Life Technologies). Data were analyzed comparing sham to BCAS-treated mice at various time points and are presented as a % of control (sham).

### Di I labeling and anastomoses measurements

To examine the cortical cerebrovasculature, sham and BCAS-treated mice were perfused with a fluorescent dye called Di I [10]. A perfusion pump (1 ml/min) was used to perfuse 5 ml of 1xPBS, immediately followed by 10 ml of Di I working solution and then 10 ml of 10% buffered formalin. Brains were carefully removed from the skull and post-fixed with 10% buffered formalin overnight at 4°C. The brains were then transferred into 1xPBS for storage, and protected from light. The whole brains were then imaged using a fluorescent microscope with a 1x objective (Nikon Eclipse E800/DS-Ri1 camera).

The number of anastomoses between the middle cerebral artery (MCA) and the anterior cerebral artery (ACA) and posterior cerebral artery (PCA) were counted in sham and BCAS-treated mice and data are presented as the total number of anastomoses between the two arteries.

### Statistical analysis

We conducted a power analysis to ensure adequate subject numbers as detailed in the figure legends for each study. All measured variables are presented as mean ± SEM from a minimum of three independent experiments. To determine significance between Sham and BCAS groups within a particular brain region (Cortex, Hippocampus, Striatum), a One-Way ANOVA was used and is defined as a *p ≤ 0.05, **p ≤ 0.01, ***p ≤ 0.001.

## Results

### Alterations within the extracellular matrix (ECM)

**Fig 2:**
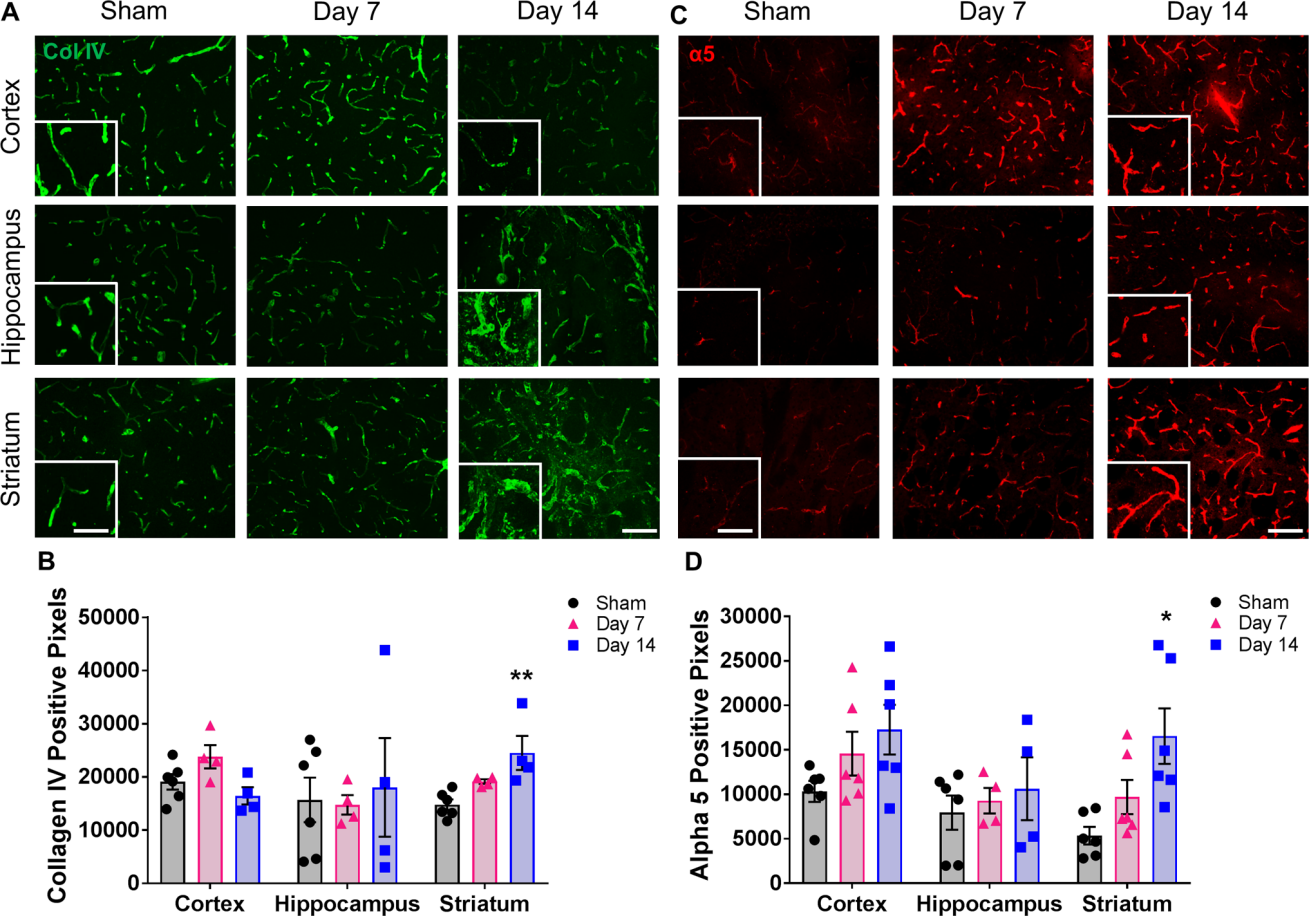
Changes in the ECM are observed in multiple regions of the brain following BCAS. **A)** Representative images of collagen IV staining (green) within the cortex, hippocampus and striatum of sham or BCAS-treated (7 and 14 days) mice. Scale bar = 100 um. Inset images are a magnified portion to show detail. Scale bar = 300 um. **B)** Quantification of collagen IV-positive pixels. N = 4–6 **p < 0.01 **C)** Representative images of alpha5 staining (red) within the cortex, hippocampus and striatum of sham or BCAS treated (7 and 14 days) mice. Scale bar = 100 um. Inset images are a magnified portion to show detail. Scale bar = 300 um. **D)** Quantification of alpha5-positive pixels. N = 4–6 *p < 0.05.

To determine if BCAS leads to alterations of the BBB-associated ECM, we examined collagen IV (a prominent vascular basement membrane component) staining in the cortex, hippocampus and striatum of sham and BCAS-treated mice (Fig 2A). No significant changes were observed in any region of the day 7 BCAS group compared to shams. However, a significant increase in collagen IV expression occurs in the striatum at day 14 (24,510 ± 4351 vs. 14,801 ± 984 pixels) compared to shams (Fig 2B). Interestingly, collagen IV staining in both the striatum and hippocampus at day 14 appeared to have an abnormal morphological expression pattern; increased thickness, irregular in shape and a disorganized pattern in some areas (Fig 2A).

Integrins are important cell surface transmembrane receptors that afford key cell-ECM interactions. In particular, the α5 integrin is conditionally expressed in cerebral blood vessels in the mature brain only after injury [11] and may play an important role in maintaining the BBB [12]. Therefore, we examined the expression of α5 integrin and found a significant increase in the striatum of day 14 BCAS-treated mice (16,548 ± 3114 pixels) compared to sham (5357 ± 982 pixels) mice (Fig 2C and 2D). While not significant, an increase in α5 expression is also observed in the cortex and hippocampus by post-stenosis day 14.

While other changes may be occurring at the day 7 time point, we were not able to detect any significant differences in components of the ECM in any brain region this early following BCAS. Therefore, all subsequent studies focused on the day 14 time point.

### Alterations within the blood-brain barrier (BBB)

**Fig 3:**
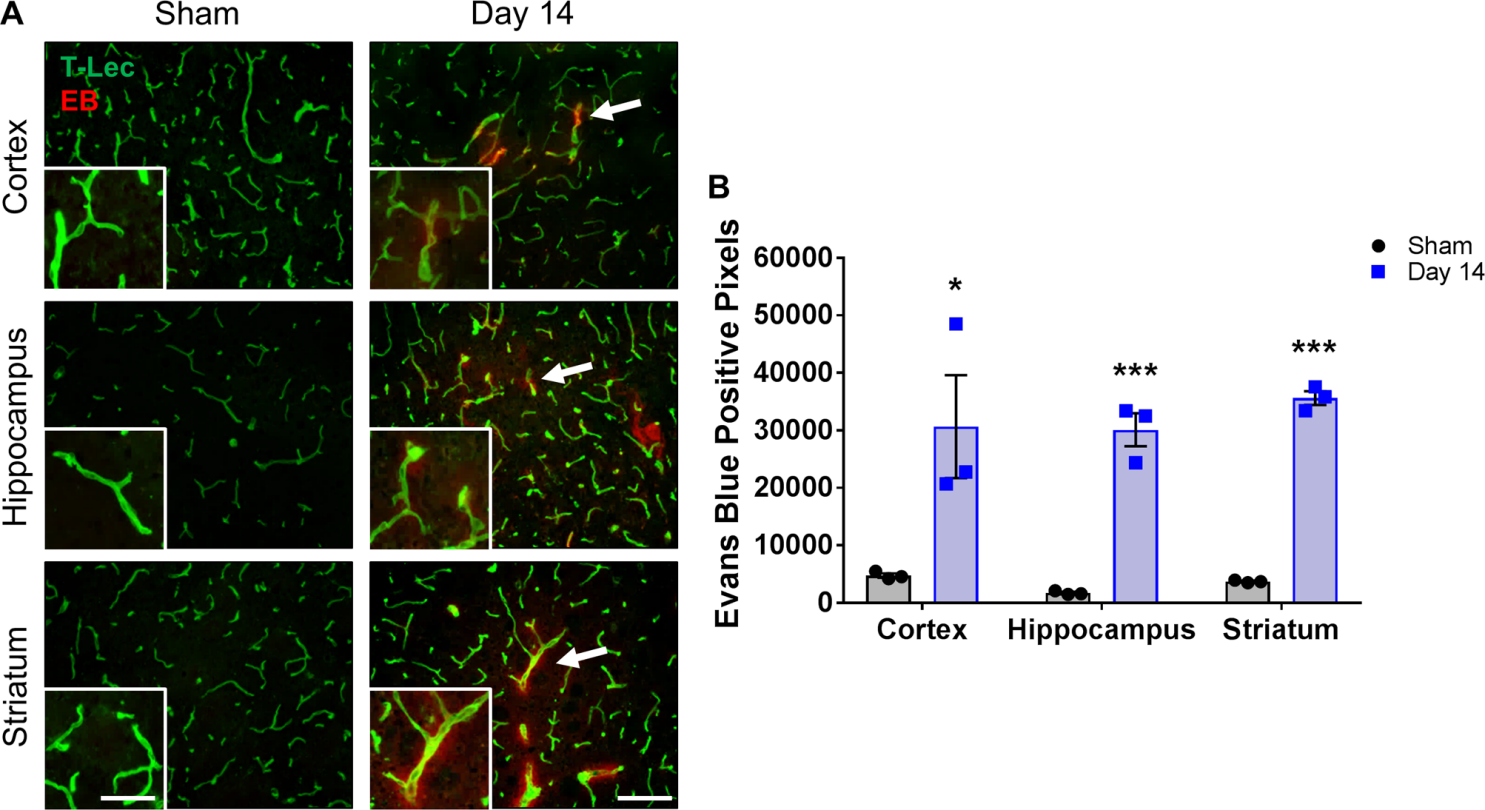
Leakage of Evans blue from blood vessels is observed in multiple regions of the brain following BCAS. **A)** Representative images of Evans blue (EB) staining (red) co-labeled with tomato-lectin (green) within the cortex, hippocampus and striatum of sham or BCAS-treated (14 days) mice. Scale bar = 100 um. Inset images are a magnified portion to show detail. Scale bar = 300 um. White arrows indicate example regions of Evans blue leakage from the endothelial cells. **B)** Quantification of Evans blue-positive pixels. N = 3 *p < 0.05, ***p < 0.001.

Leakage of substances from blood vessels into the brain parenchyma is a clear sign of BBB breakdown. Evans blue (EB) dye (fluorescent under red channel) has a high affinity for serum albumin and is a traditional marker of BBB permeability. We injected EB into sham and day 14 BCAS-treated mice and looked for extravasation within all three brain regions (Fig 3A). A significant increase in EB was detected around blood vessels (visualized by tomato-lectin staining) in the BCAS-treated group compared to the shams in the cortex (30,669 ± 8951 vs. 4766 ± 378 pixels), hippocampus (30,100 ± 2875 vs. 1729 ± 190 pixels), and striatum (35,624 ± 1205 vs. 3724 ± 128 pixels) (Fig 3B).

**Fig 4:**
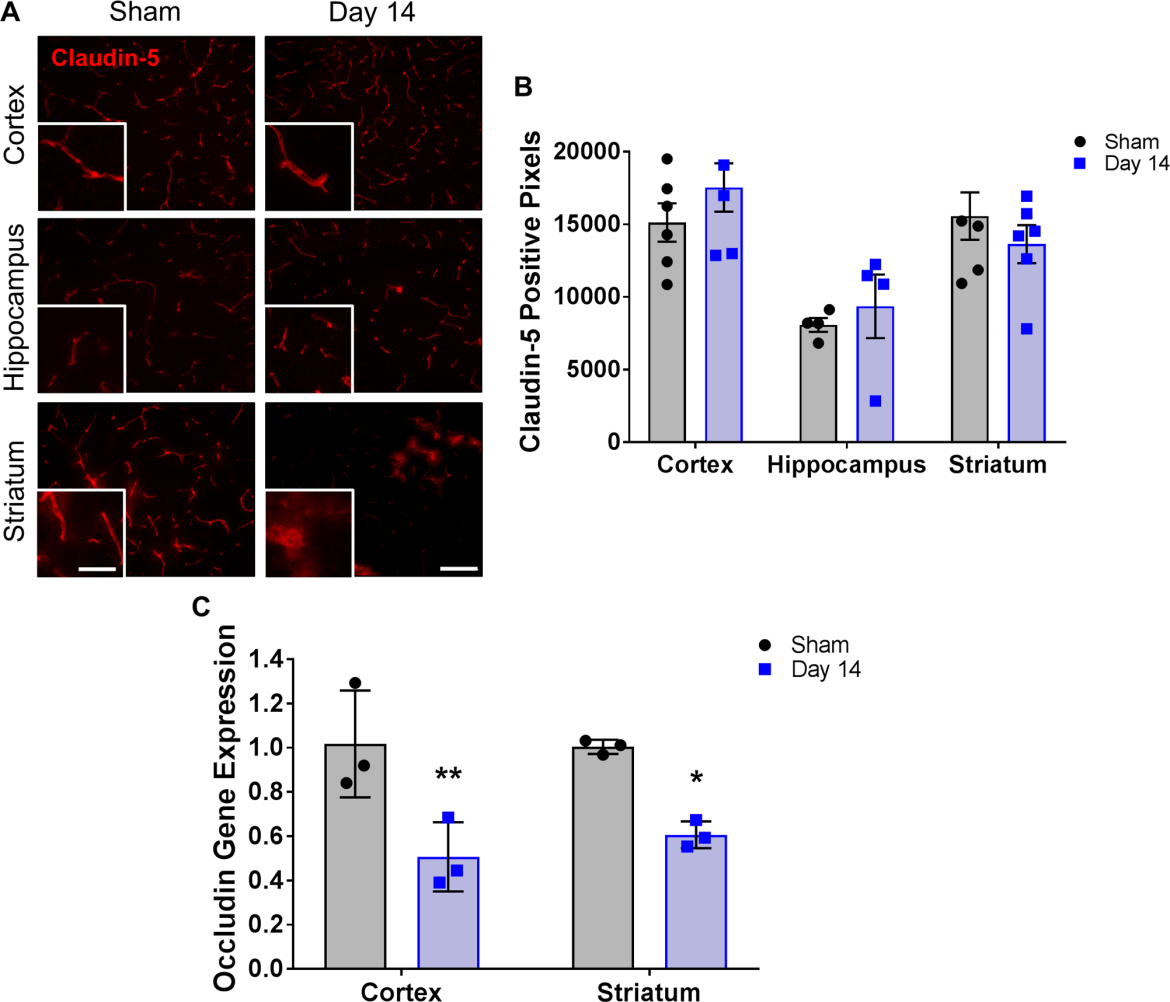
Changes in endothelial tight junctions are observed in multiple regions of the brain following BCAS. **A)** Representative images of claudin-5 staining (red) within the cortex, hippocampus and striatum of sham or BCAS-treated (14 days) mice. Scale bar = 100 um. Inset images are a magnified portion to show detail. Scale bar = 300 um. **B)** Quantification of claudin-5-positive pixels. N = 4–6 **C)** Quantification of occludin gene expression within the cortex and striatum of sham or BCAS treated (14 day) mice. N = 3 *p < 0.05, **p < 0.01.

Leakage from the blood to the brain parenchyma may be occurring due to disruptions to the tight junction proteins. In this study, total claudin-5 tight junction protein immunofluorescence does not appear to change significantly within any of the regions examined compared to shams (Fig 4A and 4B). However, its morphological expression pattern within the striatum of the 14 day BCAS-treated group appears to be altered; it appears to have lost its vascular morphology and has a less structured distribution (Fig 4A). In contrast, a second tight junction, occludin, does have significantly reduced gene expression in the cortex of the 14 day (0.51 ± 0.09 fold) BCAS-treated group compared to shams (1.0 ± 0.14 fold) (Fig 4C). A significant decrease is also observed in the striatum of the 14 day BCAS-treated group compared to shams (0.61 ± 0.04 vs. 1.0 ± 0.19 fold) (Fig 4C).

### Inflammation occurs early in BCAS

**Fig 5:**
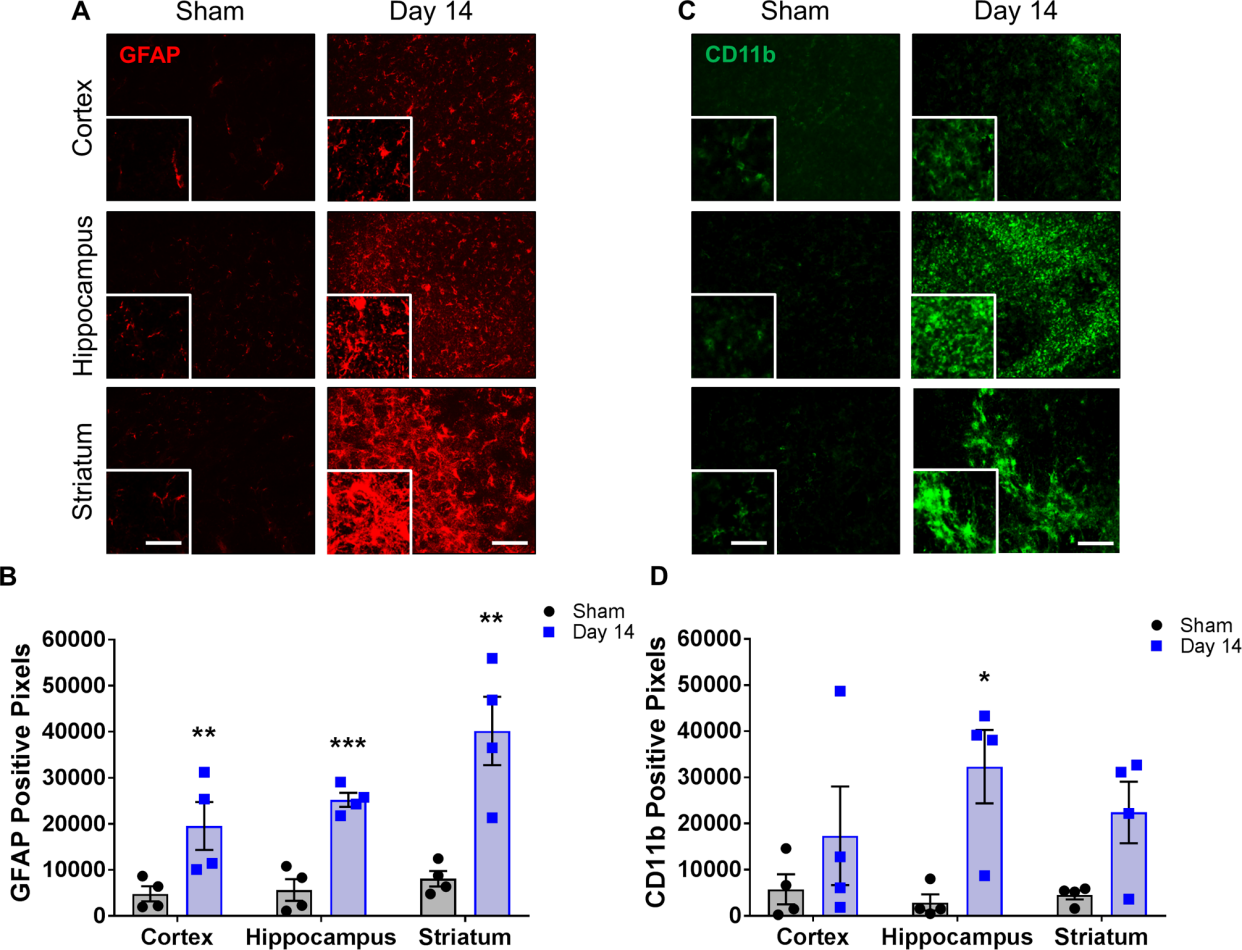
Changes in glial cells are observed in multiple regions of the brain following BCAS. **A)** Representative images of GFAP staining (red) within the cortex, hippocampus and striatum of sham or BCAS-treated (14 days) mice. Scale bar = 100 um. Inset images are a magnified portion to show detail. Scale bar = 300 um. **B)** Quantification of GFAP-positive pixels. N = 4 **p < 0.01, ***p < 0.001 **C)** Representative images of CD11b staining (green) within the cortex, hippocampus and striatum of sham or BCAS-treated (14 days) mice. Scale bar = 100 um. Inset images are a magnified portion to show detail. Scale bar = 300 um. **D)** Quantification of CD11b-positive pixels. N = 4 *p < 0.05.

Previous studies have shown an increase in inflammation within the corpus callosum at later time points following hypoperfusion. To determine if inflammation was increased at an early time point or in non-white matter tissue, we examined astrocyte and microglial expression in all three brain regions. We found a significant increase in astrocyte (GFAP) expression 14 days post-stenosis in the cortex (19,537 ± 5209 vs. 4810 ± 1635 pixels), hippocampus (25,223 ± 1521 vs. 5635 ± 2335 pixels) and striatum (40,183 ± 7430 vs. 8095 ± 1668 pixels) compared to shams, respectively (Fig 5A and 5B). We also observed increases in microglial (CD11b) expression in all brain regions at 14 days post-stenosis, with a significant increase within the hippocampus of the BCAS-treated group (32,223 ± 7952 pixels) compared to shams (2890 ± 1734 pixels) (Fig 5C and 5D).

### Increased cell proliferation in BCAS

**Fig 6:**
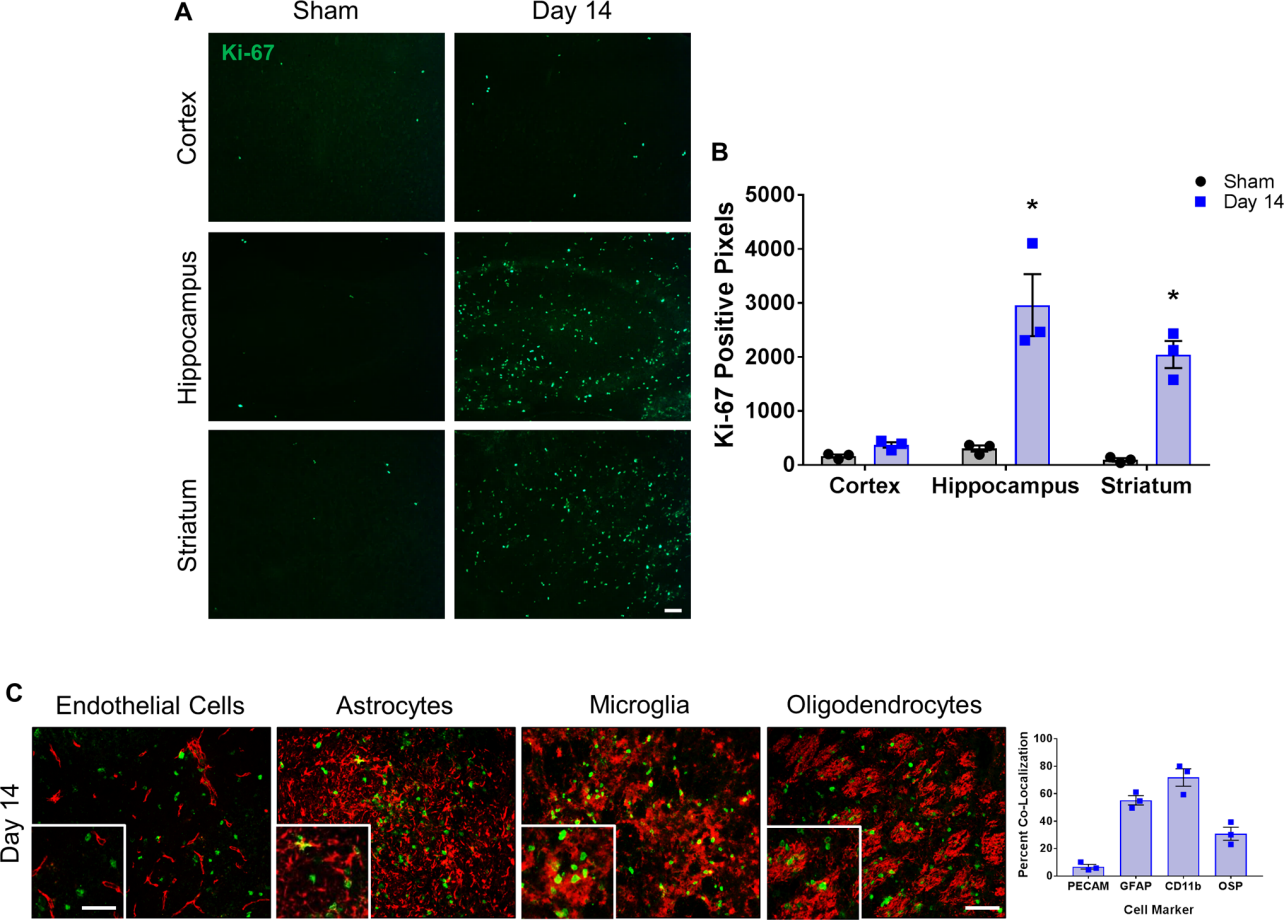
Cellular proliferation is increased in multiple regions of the brain following BCAS. **A)** Representative images of Ki-67 staining (green) and **B)** quantification of Ki-67-positive pixels in the cortex, hippocampus and striatum of sham and BCAS-treated (14 days) mice. N = 3 *p < 0.05 Scale bar = 100 um. **C)** Representative images of Ki-67 staining (green) co-labeled (red) with PECAM (endothelial cells), GFAP (astrocytes), CD11b (microglia) and OSP (oligodendrocytes) within the striatum of 14 day BCAS mice. Scale bar = 100 um. Inset images are a magnified portion to show detail. Scale bar = 300 um. Graph shows quantification of the percentage of Ki-67 positive cells co-labelled with PECAM, GFAP, CD11b or OSP from 14 day BCAS mice. N = 3.

We sought to determine whether proliferation of endothelial cells was a possible reason for the observed BBB leakage in this model, as angiogenesis is a mechanism by which the brain attempts to compensate for chronic hypoperfusion, but angiogenic endothelial cells do not form a tight BBB complex. Remarkably, we observed a significant increase in proliferating cells (labeled with Ki-67) in the hippocampus and striatum of BCAS-treated groups compared to shams (hippocampus 2958 ± 573 vs. 305 ± 56 pixels; striatum 2045 ± 250 vs. 95 ± 30 pixels) (Fig 6A and 6B). However, co-labeling of Ki-67 with PECAM-1 (endothelial marker) showed only a 6.8% co-localization and did not appear to be the primary proliferating cell type (Fig 6C). To determine which cells might be proliferating, we co-labeled Ki-67 with astrocytes (GFAP), microglia (CD11b), and oligodendrocytes (OSP) (Fig 6C). Microglia co-labelled with 71.8% of the Ki-67 positive cells, astrocytes co-labelled with 55.1% and oligodendrocytes co-labelled with 30.8% (Fig 6C). Therefore, glial cells account for the majority of the proliferating cells observed at this time point.

### Decreased number of cortical anastomoses

**Fig 7:**
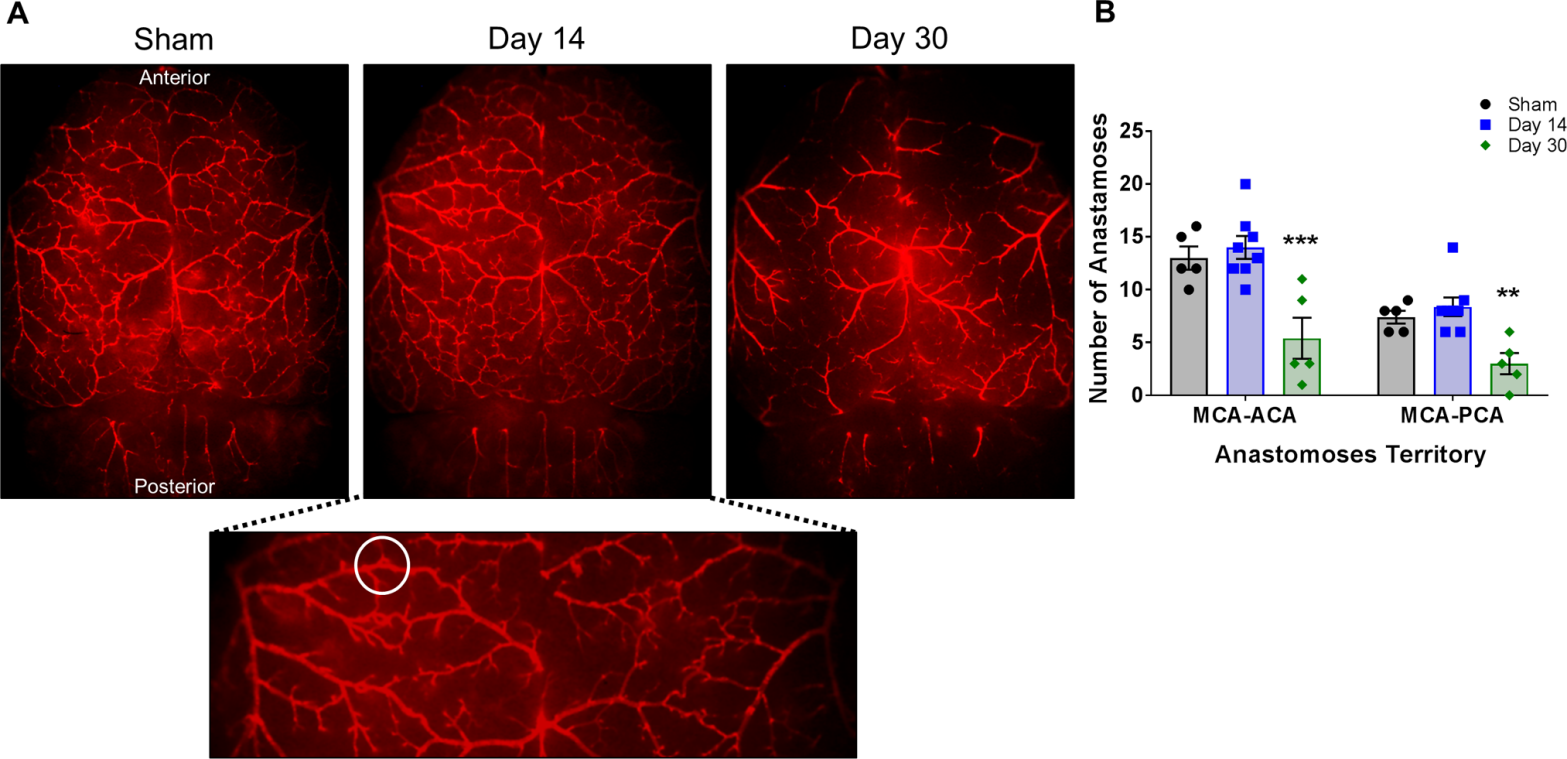
Decreased number of anastomoses in BCAS-treated mice. **A)** Representative cortical images (1x) of Di I perfused brains of sham and BCAS-treated (day 14 and 30) mice. Magnified image below to show detail. White circle is one example of an anastomosis. **B)** Quantification of the number of anastomoses between the MCA and ACA and the MCA and PCA in sham and BCAS-treated (day 14 and 30) mice. N = 5–8 **p < 0.01 ***p < 0.001 MCA: middle cerebral artery, ACA: anterior cerebral artery, PCA: posterior cerebral artery.

To examine the integrity of the cortical blood vessels which might be affected over time by BCAS, we perfused sham and BCAS-treated mice with a fluorescent Di I dye. This allowed us to visualize the branches of the middle cerebral artery (MCA), anterior cerebral artery (ACA) and posterior cerebral artery (PCA) and count the number of anastomoses (connections) between them (Fig 7). Compared to shams, the number of anastomoses within the day 14 BCAS-treated mice did not differ. In contrast, by day 30 post-stenosis (a time point commonly reported in literature) a significant decrease in the number of anastomoses between the MCA-ACA (5.4 ± 1.9 vs. 13.0 ± 1.0) and MCA-PCA (3.0 ± 1.0 vs. 7.4 ± 0.6) is observed compared to shams (Fig 7B).

## Discussion

In this study, we demonstrate that alterations in BBB permeability, BBB-associated ECM components, inflammation and cellular proliferation occur at early time points following BCAS, in both the white matter and, unexpectedly, the hippocampus and cortex. These early, non-white matter changes may contribute to the progression of further white matter damage and the resulting dementia which occurs at later time points in this model.

The brain endothelium is specialized in that it possesses features that are essential to brain homeostasis and proper cellular activities [7, 13]. This includes the specialized tight junction proteins claudin-5 and occludin. Here we report a significant decrease in occludin gene expression 14 days post-stenosis within the cortex and striatum. Toyama et al. report a decrease in claudin-5 expression in the corpus callosum 3 days post-stenosis, and equate it with a disruption in the BBB [9]. While we do not report a significant change in total claudin-5 expression based on stain-specific positive pixel density, we do note alterations in the morphological appearance of some of the claudin-5 within the striatum 14 days post-stenosis. Claudin-5 expression on blood vessels has been reported to be discontinuous following stroke [14] and has been shown to be located on a small percentage of infiltrating leukocytes in a model of multiple sclerosis, leading to the appearance of a punctate immunostaining pattern [15]. The localization change we observe went from the typical elongated blood vessel staining to one that has a more diffuse, less structured distribution (and therefore difficult to quantify), which might indicate an alteration in tight junction function since the protein may not be at the surface of the endothelial cell. The possibility exists that these endothelial cells are attempting to upregulate the expression of claudin-5 to counteract the BBB breakdown (as evidenced by Evans blue leakage); however, appropriate localization to the tight junctions is not occurring. This may lend to a lack of significant difference in total claudin-5 fluorescent levels between the sham and day 14 BCAS-treated mice.

The BBB-associated ECM plays an important role in the overall health and function of blood vessels within the brain. One of the key components is collagen IV, as it aids in the assembly of the basement membrane by creating a scaffold for other proteins such as laminin and perlecan. A decrease in the expression of collagen IV is typically observed following an injury such as stroke and is an indication of a breakdown of the BBB. In this model at these early time points, however, we do not observe a decrease in collagen IV expression. In fact, a significant increase is detected in the striatum 14 days post-stenosis. We also notice a change in the morphology of the stain in both the striatum and hippocampus, including what appears to be wall thickening. It is possible this disorganization is a secondary effect of blood vessel changes, however when we examined blood vessels using a marker of endothelial cells (PECAM-1; data not shown), we did not notice any significant morphological changes between sham and BCAS animals at these time points. Histological characterization of small vessel disease includes collagen deposition, wall thickening and smooth muscle cell degeneration [16]. Therefore, we may be observing the beginning stages of arteriopathy of small vessels within the striatum and hippocampus that is much less evident in the cortex at this early time point. Importantly, this potential vascular remodeling may also account for the changes in the staining pattern of claudin-5 in the BCAS-treated mice as discussed above.

Integrins are cell surface receptors that mediate interactions with the ECM and play important roles in survival, proliferation and differentiation [17]. The α5β1 integrin is a receptor for fibronectin, amyloid β and the domain V portion of perlecan, is upregulated only after brain injury, and has been shown to play an essential role in BBB permeability and vascular remodeling/angiogenesis following stroke [11, 12, 18–20]. For the first time, we report a significant increase in α5 expression in the striatum of BCAS-treated mice at 14 days with a trend of an increase in the cortex as well, indicating an increase in endothelial cell activation. Endothelial activation is known to lead to the loss of vascular integrity, upregulation of surface molecules, and participation in the inflammatory response and it usually has a graded response; for example, changes in endothelial cell integrity can range from small increases in local permeability to major cell contraction [21]. Therefore, even the smaller changes we observe in non-white matter regions are still an indication of unhealthy tissue.

Evans blue dye extravasation is traditionally used as a measure of BBB permeability and is of similar size to commonly used dextrans. Leakage of EB from blood vessels was observed in the cortex, hippocampus and striatum in day 14 BCAS-treated mice, suggesting a breakdown of the BBB in both white matter and non-white matter regions. The significant decrease in occludin gene expression we observe within the cortex may lead to the EB leakage observed in this region. In addition, the EB leakage within the striatum may be more severe due to the combination of decreased occludin expression and a disruption in the structure and localization of claudin-5. Miyamoto et al. report BBB leakage in the white matter, as evidenced by a significant increase in IgG staining in BCAS-treated mice at day 7 [8], though they do not report whether IgG staining was observed in non-white matter regions as well. Taken together, we report that alterations in tight junction proteins, ECM proteins and integrins, and leakage of EB occur in both white and non-white matter regions at early time points following BCAS.

Glial cells are important for BBB support and repair of injured tissues and astrocyte and microglial activation has been observed in white matter in models of hypoperfusion [9, 22–24]. This increase in glial activation has been predominantly located in the corpus callosum, though most reports do not mention examining non-white matter regions. Miki et al. have observed glial activation in grey matter regions; however this spreading to multiple regions was only reported at 35 days post-stenosis [25] and it is unclear as to when glial activation first appeared. Here we show for the first time significantly increased astrocyte and microglial activation within 14 days (as measured with immunofluorescence), not only in the striatum as expected, but also in the cortex and hippocampus, indicating injury occurs in multiple regions of the brain following hypoperfusion.

Disruption of the BBB occurs during angiogenesis, so we examined whether endothelial cells were undergoing cell proliferation following BCAS. The amount of Ki-67 (cell proliferation marker) staining was significantly increased by day 14 within the hippocampus and striatum. However, co-labeling Ki-67 with an endothelial marker showed this was not the predominately proliferating cell type; thus we co-labeled Ki-67 with markers for astrocytes, microglia and oligodendrocytes. Surprisingly, we observed some of the proliferating cells co-labeled with oligodendrocytes. The oligodendrocyte specific protein (OSP) is an important component of myelin [26] and these cells may possibly be responding to injury by upregulating OSP (also known as claudin-11) to help protect the axons from damage. However, the microglia and astrocytes appeared to account for the majority of the proliferating cells, which indicates again that inflammation is occurring at early time points after BCAS and may play an important role in injury from hypoperfusion. It is unclear whether this inflammatory reaction is beneficial or detrimental to the tissue. Studies which modulate the inflammatory pathway at early time points after BCAS are needed and may lead to novel treatments.

Since BCAS decreases blood flow to the brain, we examined the major cortical blood vessels to determine if they would be altered. Di I is a lipophilic dye that fluorescently labels endothelial cells [10] and allowed us to visualize the middle, anterior and posterior cerebral arteries. Previous studies typically report data from this model at 30 days post-stenosis or later and at this time point blood flow is decreased by approximately 25% [2, 27]. At this time point, we clearly see a significant decrease in the number of anastomoses between these major vessels. Whether portions of the vessels are no longer present or whether they are blocked and the Di I is not able to penetrate them is not known. While no difference in the number of anastomoses is observed at 14 days compared to shams, there may be ongoing processes that we are not able to detect by this method, considering the significant changes which occur by 30 days post-stenosis.

There are several limitations to this study. We set out to examine previously uncharacterized, but potentially meaningful, early changes in BCAS-treated brain tissue. While BCAS is used to model white matter injury-induced vascular dementia, it is important to note that changes are also occurring in the grey matter which may contribute to and/or worsen this white matter injury. While Evan’s blue dye is a classic method for studying BBB leakage, future studies could make use of other markers such as various sized dextrans, IgG, sodium fluorescein or biocytin, to provide more information regarding the extent of BBB permeability as well as distinguish between paracellular and transcellular BBB disruption. While difficult to perform due to the sporadic nature, quantification of the morphological alterations we observed would provide greater insight as to the cellular changes that are occurring following injury. Also, this study does not include behavioral analysis since we were looking at early time points in the disease progression; behavioral changes are not commonly reported to occur until at least 30 days post-stenosis. Future studies might include therapeutic intervention such as inhibition of alpha 5 integrin, whose absence in an alpha 5 integrin endothelial cell knockout mouse has been linked to profound post-stroke stabilization of the BBB and subsequent reduced brain injury [12]. Finally, the cell proliferation co-localization analysis requires further examination using more precise confocal imaging. While beyond the scope of this study, obtaining a better understanding of all the inflammatory pathways at play in this model could provide a potential therapeutic for this disease.

In conclusion, this study demonstrates that BCAS leads to early changes in BBB permeability and inflammation within multiple brain regions. While the overall pathology is more severe in the striatum, there are clear signs of cerebrovascular and inflammatory pathologies within the cortex and hippocampus. While each brain region responds differently to injury, it is important to note that alterations to blood vessels and blood flow do affect all brain regions at various time points. The changes observed here, along with alterations in large surface blood vessels, might underlie the gradual development of white and non-white matter pathology and dementia associated with this model suggesting that therapies that target such BBB instability or inflammation might be beneficial as early intervention.

## Supporting information

S1 File The ARRIVE guidelines checklist. (PDF)
